# Increasing Access to Tuberculosis Services in Ethiopia: Findings From a Patient-Pathway Analysis

**DOI:** 10.1093/infdis/jix378

**Published:** 2017-11-06

**Authors:** Lelisa Fekadu, Christy Hanson, Mike Osberg, Julia Makayova, Pia Mingkwan, Daniel Chin

**Affiliations:** 1 Federal Ministry of Health, Tuberculosis and Leprosy Disease Prevention and Control Team, Addis Ababa, Ethiopia;; 2 Macalester College, St. Paul, Minnesota; and; 3 Bill and Melinda Gates Foundation, and; 4 Linksbridge, Seattle, Washington

**Keywords:** Tuberculosis, patient-pathway analysis, health extension workers, care seeking, Ethiopia

## Abstract

**Background:**

In Ethiopia, extensive scale-up of the availability of health extension workers (HEWs) at the community level has been credited with increased identification and referral of patients with presumptive tuberculosis, which has contributed to increased tuberculosis case notification and better treatment outcomes. However, nearly 30% of Ethiopia’s estimated 191000 patients with tuberculosis remained unnotified in 2015. A better understanding of patient care-seeking practices may inform future government action to reach all patients with tuberculosis.

**Methods:**

A patient-pathway analysis was completed to assess the alignment between patient care initiation and the availability of diagnostic and treatment services at the national level.

**Results:**

More than one third of patients initiated care with HEWs, who refer patients to health centers for diagnosis. An additional one third of patients initiated care at health centers. Of those health centers, >80% had microscopy services, but few had access to Xpert. Despite an extensive microscopy and radiography network at middle levels of the health system, a quarter of all notified patients with tuberculosis had no bacteriological confirmation of disease. While 30% of patients reported receiving some form of care from the private sector, private-sector facilities, especially pharmacies, were not widely accessed for tuberculosis diagnosis.

**Discussion:**

The availability of HEWs can increase access to tuberculosis diagnostic and treatment support services, particularly for rural populations. Continued strengthening of referral systems from HEWs and health posts are needed to enable consistent and timely access to Xpert as an initial diagnostic test and to drug resistance screening.

Ethiopia is the second-most-populous country in Africa and has a distinctly young population; 45% of Ethiopians are aged <15 years [1]. In the past decade, Ethiopia has made significant gains in socioeconomic development, achieving a 33% reduction in poverty between 2000 and 2011 [1]. Efforts to improve healthcare access for the rural majority have ensured that >90% of the population lives within 5 km of a healthcare provider. Furthermore, Ethiopia has almost achieved equity in healthcare utilization across all wealth quintiles. Over the past decade, Ethiopia has reduced its mortality rates among children aged <5 years, infants, and neonates by 47%, 39%, and 25%, respectively [2].

While Ethiopia has made remarkable strides, the World Health Organization (WHO) estimates that in Ethiopia, 60%–80% of early mortality is due to preventable communicable diseases such as malaria, pneumonia, and tuberculosis [3]. Tuberculosis remains the third greatest cause of death in Ethiopia [4]. Over 60% of all notified tuberculosis cases in Ethiopia occur among people ≤35 years of age [4]. The concentration of the tuberculosis burden for young Ethiopians could present barriers to the country’s economic and social development. Coinfection and drug resistance continue to pose risks to the population, with an estimated 8% of patients with tuberculosis living with human immunodeficiency virus and 2.7% of new patients presenting with multidrug-resistant tuberculosis [3, 5].

Since a peak in 1996, prevalence rates of tuberculosis in Ethiopia have declined by >50%. The WHO estimated that there were 191000 new cases of tuberculosis in 2015 [3]. From 2005 to 2015, Ethiopia improved the treatment success rate from 79% to >90%, increased the case detection rate from 33% to 71%, and lowered tuberculosis mortality from 73 to 30 cases per 100000 [6]. However, nearly 30% of incident cases remained missing (ie, undiagnosed or unreported) in 2015 [3]. Moreover, <20% of the estimated 3300 Ethiopian patients with multidrug-resistant tuberculosis commenced treatment in 2015 [3].

Ethiopia is divided into 9 regions and 2 administrative city councils, Addis Ababa and Dire Dawa. Approximately 76% of Ethiopians live in rural areas, with 8% belonging to pastoralist communities [1]. Ethiopia’s primary healthcare unit has a 3-tiered healthcare delivery system. The first, the *woreda* (ie, district) health system, comprises a primary hospital (with a catchment population of 60000–100000), health centers (1 facility per 15000–25000 population), and their satellite health posts (1 facility per 3000–5000 population), all of which are linked through referral systems. The second tier comprises a general hospital (with a catchment population of 1 million–1.5 million). The third tier comprises a specialized hospital with a catchment population of 3.5 million–5 million.

In 2004, the Government of Ethiopia introduced the Health Extension Program, a free primary healthcare package that includes disease prevention and control, family health services, hygiene and environmental sanitation services, and health education and communication [7]. Alongside the Health Extension Program, the Government of Ethiopia introduced a new cadre of community health workers, called health extension workers (HEWs). HEWs and the flagship Health Extension Program constitute the backbone of health service delivery at the community level in Ethiopia. HEWs receive 1 year of training in basic health service delivery and are salaried government employees [8]. In 2016, there were >38000 HEWs employed in Ethiopia [8]. In a hub and spoke model, each community has its own health post linked to a network of HEWs. The HEWs spend 25% of their time in the health post and 75% of their time working in the community [8]. HEWs provide tuberculosis education, referral, and treatment follow-up. The engagement and accessibility of HEWs likely contributed to a significant increase in health service coverage in recent years and, more specifically, to many of the recent gains made in tuberculosis care [3].

The success of HEWs in Ethiopia underlines the importance of understanding how patients interact with the health system. Studying how and where patients access care creates opportunities to optimize the use of the existing health network and to strategically plan for expansion of service availability such that tuberculosis diagnostic and care services better meet patients where they are.

## METHODS

The main objective of this patient-pathway analysis (PPA), the method of which is described by Hanson et al [9] elsewhere in this supplement, was to assess the alignment between patient care initiation and the availability of diagnostic and treatment services at the national level. The PPA for Ethiopia was completed at the national level, using data sources from 2011, 2014, and 2016. The data sources for each column in the analysis are shown in Table 1.

**Table 1.** Data Sources for the Patient-Pathway Analysis (PPA)

**PPA Component**	**Data Source**
Care seeking for general illness	2014 Household Health Service Utilization and Expenditure Survey [2]
Tuberculosis diagnostic and treatment services coverage	2016 Services Availability and Readiness Assessment [10]
Tuberculosis treatment location	2011 Tuberculosis Prevalence Survey [5]
Tuberculosis case notification and treatment success	2016 World Health Organization Global Tuberculosis Database [3]

Since each data source uses a different naming convention for health facilities, all facilities were categorized as either public, private (formal), or private (informal) and as belonging to one of the 4 levels in the health system, to allow for comparison.

The health facilities were defined by the following health levels. Level 0 (L0) refers to the most basic care level, which is usually community based. Services at L0 facilities include basic triage, provision of health information, and essential prevention activities and care. Services are commonly provided as an extension of facility-based care and are provided by volunteers or paramedical staff with limited formal training. No laboratory testing is available, but L0 staff may serve as treatment supporters for patients with tuberculosis. Examples of facilities and personnel are health posts and HEWs (public) and pharmacies and traditional healers (private). Level 1 (L1) facilities provide primary healthcare. Nurses, midwives, or private physicians commonly provide L1 services, generally on an outpatient basis. Some basic diagnostic services and essential medicines may be available. Examples are health centers (public) and clinics (private). Level 2 (L2) facilities provide primary healthcare, as well as more-advanced care. L2 facilities commonly have more-extensive diagnostic and treatment options and can provide both outpatient and inpatient care. Examples of facilities are primary and general hospitals (public) and nongovernmental organization facilities, and private hospitals (private). Level 3 (L3) facilities provide specialized care with a large inpatient capacity. L3 facilities provide access to specialized physicians and have more-sophisticated diagnostic and treatment capabilities. Table 2 provides a detailed mapping of the health facilities from each data source to the standard categories described above.

**Table 2.** Health Facility Coding

**Data Source, Health Facility Type**	**Categorization**	
	**Health Facility Sector**	**Health Facility Level**
2011 Tuberculosis Prevalence Survey [5]		
Government hospital	Public	2
Health center	Public	1
Health post	Public	0
Private hospital	Private	2
NGO facility	Private	1
Private clinic	Private	1
Pharmacy	Informal private	0
Other	Other	Other
NA	NA	NA
2014 Household Health Service Utilization and Expenditure Survey [2]		
Hospital	Public	2
Health center	Public	1
Health post	Public	0
*Kebele* health worker facility	Public	0
Hospital	Private	2
Clinic	Private	1
Employer organization clinic	Private	1
Health center	Private	1
NGO facility	Private	1
Pharmacy	Informal private	0
Traditional and religious healer facility	Informal private	0
Mobile HIV testing facility	Other	0
Not stated	Other	Other
2016 Services Availability and Readiness Assessment [10]		
Referral hospital	Public	3
General hospital	Public	2
Primary hospital	Public	2
Health center	Public	1
Higher clinic	Private	2
Lower clinic	Private	1
Medium clinic	Private	1

Abbreviations: HIV, human immunodeficiency virus; NA, not applicable; NGO, nongovernmental organization.

**Figure 1.**
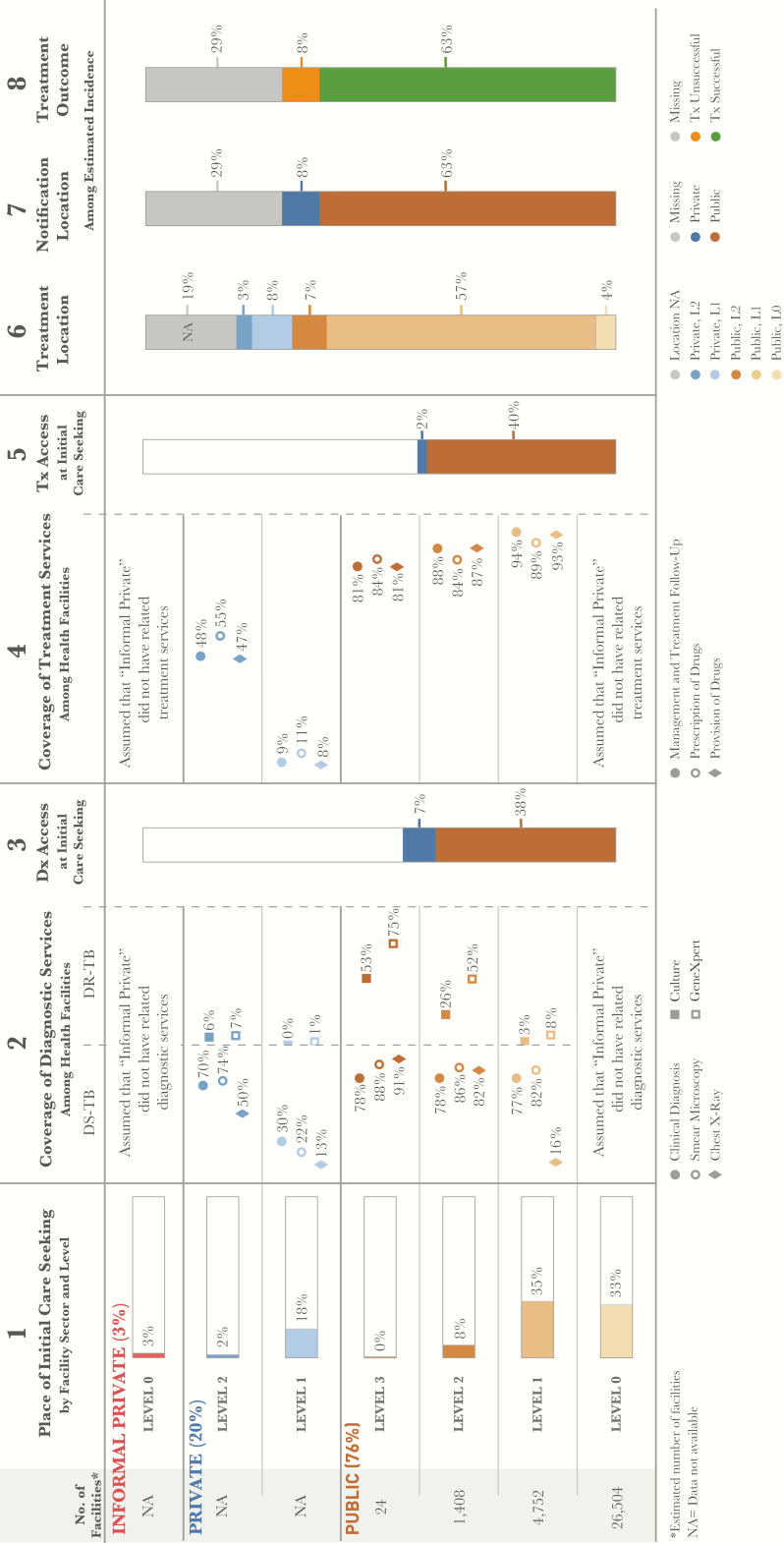
Patient-pathway visual at the national level. The patient pathway describes the care-seeking patterns of patients and how those patients may intersect with tuberculosis services. Column 1 starts by showing the sectors and levels of the health system (sectors and levels where no data was available are not included in the pathway). The percentage next to each sector title is the share of patients who initiate care seeking in this sector [2]. Next is the estimated number of health facilities at each level within each sector. There are no data available for the number of health facilities in the private sector (formal or informal). The public-sector numbers are estimated using guidelines for the number of patients each type of facility is intended to cover [2]. The final part of column 1 depicts the percentage of patients seeking initial care, by level [2]. Column 2 shows the estimated percentage of health facilities that have tuberculosis diagnostic tools at each sector and level of the health system [10]. Diagnostic tools are separated by tools for diagnosing drug-susceptible tuberculosis and those diagnosing drug-resistant tuberculosis. Column 3 shows the estimated percentage of patients likely to access a facility with tuberculosis diagnostic tools available on their initial visit to a healthcare facility. Access to diagnosis was calculated by multiplying care seeking at each level by a metric from the 2016 Services Availability and Readiness Assessment that estimated overall diagnostic coverage at each sector and level. Columns 3 and 5 separate public and private sectors on the basis of each sector’s contribution to tuberculosis services access at initial care seeking. Column 4 shows the estimated percentage of health facilities that have tuberculosis treatment services at each sector and level of the health system [10]. Column 5 shows the estimated percentage of patients likely to access a facility with tuberculosis treatment provision services available on their initial visit to a healthcare facility (calculated by multiplying column 1 and column 4). Column 6 shows the location of treatment among participants in the 2011 prevalence survey who had previously received tuberculosis treatment [5], with the assumption that this metric provides the most representative picture of where patients are treated nationwide. Treatment received in the informal private sector (<1%) and at facilities categorized as “other” (<2%) are excluded from this column. Column 7 shows which sector provided case notification, and values are calculated as a share of the overall estimated incidence in 2015 [3]. Column 8 shows the treatment outcome of notified cases among the overall estimated incidence for 2015 [3]. Columns may not add to 100%, owing to rounding. For more details on data sources used in the pathway, see the Supplementary Materials. Abbreviation: NA, not available.

The Ethiopian Ministry of Health provides estimates on the size of the population that each health facility is intended to serve. These estimates were used to estimate the total number of health facilities at each level in the public sector (column 1, Figure 1). The location of care initiation reflects data from the 2014 National Household Health Service Expenditure and Utilization Survey (HHSEUS) [11]. Specifically, data were included from survey respondents who sought care for general illnesses in outpatient services. These estimates are shown in column 1 of the patient-pathway visual (Figure 1). General care-seeking data were used for the national analysis, owing to a paucity of data on care-seeking patterns among individuals with tuberculosis symptoms.

A 2016 Services Availability and Readiness Assessment (SARA) [10] provided estimates of the percentage of health facilities that had tuberculosis diagnostic tools available in both the private and public sectors. The survey included the following diagnostic services: diagnosis on the basis of clinical symptoms, smear microscopy, culture, radiography, and Xpert. These estimates are shown in column 2 of the PPA (Figure 1).

Column 3 of the PPA shows the estimated likelihood that a patient will encounter a tuberculosis diagnostic service at the point of care initiation (Figure 1). This value was calculated by multiplying the percentage of patients who sought care at a given health facility sector and level by the percentage of facilities at that level with any of the diagnostic tools shown in column 2.

The data on treatment coverage in each facility (column 4, Figure 1) were derived from the 2016 SARA report and included treatment services such as provision of tuberculosis drugs, prescription of tuberculosis drugs, and management and treatment follow-up for patients with tuberculosis. Column 5 shows the estimated likelihood that a patient initiated care in a facility that could provide antituberculosis drugs (Figure 1). As with column 3, the estimated likelihood that a patient initiated care in a facility that could provide antituberculosis drugs was calculated by multiplying the percentage of patients who were treated at a given health facility sector and level by the percentage of facilities at that level with the capacity for drug provision (column 4, Figure 1). Information about the location where patients with tuberculosis received treatment was provided by the 2011 National Tuberculosis Prevalence Survey, represented in column 6 of the PPA (Figure 1).

The final 2 columns of the PPA show the percentage of notified tuberculosis cases and the treatment outcomes of these cases (Figure 1). The data for both columns came from the 2016 WHO global tuberculosis database. Column 7 shows the percentage of all estimated incident cases that were notified to the National Tuberculosis Program, as well as those that were missing. The notified cases were broken down into 2 categories based on location of notification: cases notified by the public sector and those notified by the private sector. Finally, column 8 provides the percentage of all estimated patients with tuberculosis who were successfully treated, based on the treatment success rate of 89% among notified cases.

When interpreting the patterns of care seeking, it is important to note that the data come from a national HHSEUS, which reports the location of care initiation for general outpatient care. While this serves as a useful proxy for tuberculosis care initiation, there may be subnational locations where there are important differences in care initiation for tuberculosis and care initiation for general illness.

### Limitations

There are several important limitations to consider when interpreting the PPA. First, it uses general care-seeking data as a proxy for care seeking by patient with tuberculosis symptoms, owing to lack of data for the latter group. Our assumption is that before patients receive a diagnosis, they will tend to follow similar initial care patterns for most common symptoms (eg, cough, fever, and mild pain). It would be instructive to test this assumption in further studies. Second, the PPA looks primarily at coverage of tools (eg, diagnosis and treatment) within health facilities and calculates a metric for access, based on that coverage. However, this does not factor in the reliability and quality of those tools nor does it incorporate the capacity for staff to use these tools appropriately. In places where quality, reliability, or capacity may be lacking, these coverage metrics may be lower. Finally, the PPA uses several data sources to estimate what a patient’s journey through tuberculosis care might look like. To limit potential data differences due to the different times of the data sources, we only use sources from 2005 onward. While only a prospective cohort study could capture the actual pathway of patients through the tuberculosis system, we have found that, by using readily available data, a programmatically useful estimation can be made by the PPA methods. Further limitations of the PPA methods are described elsewhere [9].

## RESULTS

### The Majority of Care Initiation Occurred in The Public Sector, With One Third Initiating Care With a Health Extension Worker

Commensurate with the dominance of public-sector facilities in Ethiopia, 76% of patients initiated care in the public sector, 22% initiated care in the formal private sector, and approximately 3% initiated care with traditional healers or pharmacies (Figure 1). Overall, L1—representing public health centers and private clinics—was the most utilized level of care, hosting 53% of all initial care visits.

More than one third of patients initiated care with HEWs. While tuberculosis diagnostic technologies were not available at the community level, all patients presumed to have tuberculosis should have been referred to a corresponding health center by HEWs, according to the standard scope of work for HEWs. At the health center level (L1), referred patients joined the 35% of patients who initiated care at this level. Smear microscopy was available in 82% of health centers. However, only 16% of L1 public facilities had radiography, and <5% had Xpert machines. At L2 in the public sector, approximately 80% of facilities had radiography, and >50% had Xpert. In the private sector, where 20% of patients sought care, diagnostic availability was lower than in the public sector. Approximately 22% of private clinics offered smear microscopy, 13% provided radiography, and only 1% had Xpert machines. Hospitals, in both the private and public sectors, had the highest coverage of all diagnostic tools, although <10% of all patients initiated care in these facilities. Because the majority of patients initiated care with HEWs or in facilities with lower diagnostic coverage, the proportion of patients with presumptive tuberculosis who accessed a diagnostic technology at the site of care initiation was 45%. If one assumes that all patients who accessed an HEW were referred to a health center, patient care initiation—including the referral process from HEWs to health centers—would yield access to a diagnostic technology in approximately 61% of cases.

### Most Facilities in the Public Sector Offered Tuberculosis Treatment

The vast majority of public-sector facilities had the capacity to treat tuberculosis. In the private sector, treatment was available in >50% of hospitals, although private hospitals represented the site of care initiation for only 2% of patients. Tuberculosis treatment was available in <11% of the private clinics, where 18% of patients initiated care. While HEWs provided treatment support, they did not initiate treatment or prescribe antituberculosis medicines. As such, they do not appear in the pathway as treatment providers. Excluding HEWs as treatment sites, 42% of patients accessed tuberculosis treatment at the site of care initiation. Considering that HEWs may support tuberculosis treatment where their corresponding health centers initiate treatment and have medicines available, it is estimated that approximately 62% of patients were able to receive treatment where they initiated care.

Among the patients with antituberculosis treatment history in the tuberculosis prevalence survey [5], nearly 70% reported that they received tuberculosis treatment in the public sector. This corresponds nearly exactly to the proportion of all estimated patients with tuberculosis who were notified in 2015 and is close to the 76% of patients who initiated care in the public sector. However, while 22% of care initiations occurred in private facilities, only 11% of patients with confirmed tuberculosis reported being treated there.

## DISCUSSION

With 76% of initiating care initiated in the public sector, programmatic changes adopted by the National Tuberculosis Program and implemented across the primary care network can be expected to yield population-level results. The PPA points to a need for modest changes to tuberculosis diagnosis and care—changes that stand to rapidly accelerate case detection and subsequent treatment.

The widespread patient use of HEWs reflects how valuable this cadre of health personnel is, especially where they have improved access to health services for the rural majority. Among tuberculosis cases notified in 2016, approximately 38% were referred from HEWs and health posts. Meanwhile, the data suggested that 68% of patients with presumptive tuberculosis initiated care in these facilities. This suggests an important drop-off of patients between their initial access to care and the point of accessing a diagnosis. This represents a potential source of missing cases. While the PPA did not document the extent to which patients delayed diagnosis and treatment from the time of a referral from a L0 facility, other studies have suggested that there are socioeconomic and geographic barriers to accessing higher-level care, as would be needed to follow through on a referral.

Similarly, the magnitude of clinical diagnoses (23% of notified cases) as compared to bacteriologically confirmed cases (27%) may also point to challenges in accessing diagnostic technology [3]. The near-term future of tuberculosis control will see expanded availability of Xpert across at least public L2 facilities and, possibly, Xpert Omni at L1 facilities. The diagnostic process will continue to require access to technologies that are available at levels higher than L0. Sputum collection by HEWs and specimen transport systems from HEWs to equipped laboratories may be more efficient than the continued transfer of patients. Efficiency gains would include patient savings, given the absence of indirect costs that could be associated with traveling to a higher-level facility and the avoidance of delays that could emerge with the need to travel to seek higher-level care. Strengthening systems of specimen capture, rapid sputum transport, and testing of patients who initiate care at L0 facilities may reduce the lag between the presentation of a patient presumed to have tuberculosis and diagnosis of tuberculosis for that individual. Further operational research to better understand the barriers to acting on a referral and to evaluate the impact of solutions may be useful to inform programmatic priorities.

Similarly, unless radiography and Xpert (possibly Omni) become more routinely available at L1 facilities, systematic referral of patients or specimens between L1 and L2 facilities is needed. Results from the national prevalence survey indicated that microscopy was not a particularly sensitive test and therefore incorrectly dismissed many patients with tuberculosis. Radiography, while not particularly specific, was demonstrated to be highly sensitive, rendering it an effective screening tool. Given the limited availability of radiography below L2 facilities in the public sector and the limited utilization of L2 facilities as an initial point of care, it is likely that many cases of tuberculosis are missed on the basis of the nearly exclusive use of microscopy for diagnosis. The National Tuberculosis Program may need to consider the use of digital radiography for screening as part of a new diagnostic algorithm that both maintains the patient-centered aspects of the Ethiopian health system and expands access to sophisticated diagnostic technologies.

The PPA made use of various sources of existing data, ranging from the 2011 National Tuberculosis Prevalence Survey to the 2016 Service Availability and Readiness Assessment. Changes in the health system, such as the expansion of the HEW population and the increased availability of tuberculosis diagnostic technologies, may have impacted patient care-seeking behavior. It would be useful to repeat the PPA with data from patients with tuberculosis and patients with presumptive tuberculosis to update the data on care-seeking patterns among patients with tuberculosis and their alignment with the locations of diagnosis and treatment.

## Supplementary Data

Supplementary materials are available at *The Journal of Infectious Diseases* online. Consisting of data provided by the authors to benefit the reader, the posted materials are not copyedited and are the sole responsibility of the authors, so questions or comments should be addressed to the corresponding author.

## Supplementary Material

Supplementary Appendix

## Notes

***Supplement sponsorship.*** This work is part of a supplement sponsored by the Bill and Melinda Gates Foundation.

***Potential conflicts of interest.*** All authors: No reported conflicts of interest. All authors have submitted the ICMJE Form for Disclosure of Potential Conflicts of Interest. Conflicts that the editors consider relevant to the content of the manuscript have been disclosed.
