# Supplementary material for: Increasing Access to Tuberculosis Services in Ethiopia: Findings From a Patient-Pathway Analysis
Source: J Infect Dis. 2017 Nov 6;216(Suppl 7):S696–701. doi: 10.1093/infdis/jix378 (PMC5853928; doi:10.1093/infdis/jix378)
Supplement: Supplementary Appendix [file jix378_suppl_supplementary_appendix.docx]

| **Country: Ethiopia** | | | | | |
| --- | --- | --- | --- | --- | --- |
| **Data Source** | **Survey Type** | **Survey Question** | **Reported Metric** | **Sample Size** | **PPA Step** |
| 2014 Household Health Service Utilization and Expenditure Survey | Nationally representative cross-sectional sample survey of n = 10,060 households drawn from all regional states. | 5.2 Choice of Outpatient Service Providers  “At the national level, the main provider of outpatient services was government health facilities (76.52%), followed by private health facilities (19.8%), traditional and religious healers (2.13%), and NGOs (1.38%).” (p.41) | Outpatient health care seeking behavior  (Table 19) | N = 10,060 households surveyed; Report does not provide ns for individual question responses | Step 1 –  Initial care seeking patterns |
| 2016 Services Availability and Readiness Assessment  TB section of SARA report is Chapter 3.5 (pp. 79-85) | Nationally representative cross-sectional sample survey of n = 705 health facilities, with an oversampling of hospitals. | “Diagnosis of TB through X-ray (16 percent) and of MDR-TB through rapid test (GeneXpert MTB/RIF) (6 percent) is services provided by the health facilities at low scale.” (p.79) | Percentage of facilities that offered TB diagnostic testing (clinical symptoms, smear microscopy, culture, rapid test, chest x-ray)  (Table 3.5.1) | N = 547 facilities that offer TB services | Step 2 – Diagnostic coverage |
|  |  | “Table 3.5.1 shows the percentage of facilities offering tuberculosis services by Facility type, Managing authority and Urban/Rural. Overall, 63 percent of health facilities offered TB services, while 54 percent of the health facilities provide management and treatment follow-up for TB patients.” (p.79) | Percentage of facilities that offered TB treatment coverage (prescription of TB drugs, provision of TB drugs, management and treatment follow up)  (Table 3.5.1) | N = 547 facilities that offer TB services | Step 4 – Treatment coverage |
| 2011 Tuberculosis Prevalence Survey | Nationally representative sample of 46,697 adults age > 15 years old in 85 clusters throughout the country, stratified in rural, urban, and pastoralist areas. | 7.5 Where are/were you getting TB treatment? (most recent episode) (One answer only)  1. Government Hospital (7.0%)  2. Health Center (56.8%)  3. Health Post (4.2%)  4. Private hospitals (3.0%)  5. Private Clinic (6.6%)  6. Pharmacy (.3%)  7. NGOs (1.8%)  8. Traditional Healers  9. Other (1.2%)  (Source: Annex H Form 5 Re-interview, p.92) | Place where respondent previously received TB treatment  (Table 9) | N = 46,697 adults age > 15 years old surveyed;  N=733 respondents that reported anti-TB treatment in last 5 years | Step 6 –  TB treatment location |
| 2016 WHO Global TB Report | Annual report providing data on TB epidemiology, health systems and financing for 194 member state.  Notification location data available in table 4.2 of annual report.  Raw data accessed via Global TB Database available here:  <http://who.int/tb/data/en/> | N/A | Estimated burden – 191,000  TB treatment coverage (case detection rate) – 71%  New and relapse notified cases –  135,951  Share of notified cases from private sector – 11%  Treatment success rate – 89% | | Step 7 –  Among estimated burden-notification source  +  Step 8 –  Among estimated burden-successfully treated cases |
